# Lipotoxicity and immunometabolism in ischemic acute kidney injury: current perspectives and future directions

**DOI:** 10.3389/fphar.2024.1355674

**Published:** 2024-02-23

**Authors:** Afolarin A. Otunla, Kumaran Shanmugarajah, Alun H. Davies, Joseph Shalhoub

**Affiliations:** ^1^ Department of Surgical Biotechnology, University College London, London, United Kingdom; ^2^ Cleveland Clinic, Cleveland Clinic Transplant Centre, Cleveland, OH, United States; ^3^ UK and Imperial Vascular Unit, Section of Vascular Surgery, Department of Surgery and Cancer, Imperial College London, Imperial College Healthcare NHS Trust, London, United Kingdom

**Keywords:** ischemia, lipotoxicity, immunometabolism, acute kidney injury, mitcohondria

## Abstract

Dysregulated lipid metabolism is implicated in the pathophysiology of a range of kidney diseases. The specific mechanisms through which lipotoxicity contributes to acute kidney injury (AKI) remain poorly understood. Herein we review the cardinal features of lipotoxic injury in ischemic kidney injury; lipid accumulation and mitochondrial lipotoxicity. We then explore a new mechanism of lipotoxicity, what we define as “immunometabolic” lipotoxicity, and discuss the potential therapeutic implications of targeting this lipotoxicity using lipid lowering medications.

## Introduction

Lipotoxicity refers to the “accumulation of excessive lipids in non-adipose tissues, leading to cell dysfunction or death” (Schaffer, 2003). Lipid accumulation within the constituent cells of the kidney is well documented in a range of aetiologies of acute kidney injury (AKI) (Tannenbaum et al., 1983; Zager et al., 2001; Zager et al., 2005). Whether this lipid accumulation leads to “cell dysfunction or death” was initially a matter of debate. Some argued that lipid accumulation was simply a consequence of renal injury, with the subsequent reduction in mitochondrial aerobic respiration leading to decreased fatty acid oxidation, redirection of free fatty acids (FFAs) to triglyceride storage and lipid accumulation (Ruidera et al., 1988). However, a series of animal experiments across the last 3 decades has generated a substantial pool of evidence to suggest that excess renal lipids are nephrotoxic (Matthys et al., 1984; Bernardi et al., 2002; Schönfeld and Wojtczak, 2008; Katsoulieris et al., 2010; Müller et al., 2017).

The emergent school of thought identified FFAs as the principal determinant of lipotoxicity, with accumulation leading to mitochondrial dysfunction, reactive oxygen species generation and cell death through activation of apoptotic pathways (Erpicum et al., 2018). These “direct” mechanisms of lipotoxicity could be the tip of the iceberg, as it is becoming increasingly clear that FFAs are actively involved in the immune response. FFAs are able to stimulate immune cell activation and development via pattern recognition receptors (PRRs), fine tuning the immune response to the local metabolic microenvironment (Radzikowska et al., 2019; Morgan et al., 2021). Conversely, activation of PRRs can trigger long term changes to immune cell lipid metabolism. This interplay between the immunologic and the metabolic is known as ‘immunometabolism’ and is a fundamental aspect of the coordinated response to injury.

The kidney is uniquely placed at the interface of the metabolic and the immune due to its high metabolic rate and exposure to high concentrations of metabolic waste products, cytokines and various antigens. Despite this, the role of immunometabolic pathways in AKI is not well characterised or understood.

The studies which do exist point towards proximal tubular epithelial cells (PTECs) as a central mediator (van der Rijt et al., 2022). From a metabolic standpoint, the reasoning behind this is clear; PTECs are crucial in maintaining renal metabolic homeostasis, driving reabsorption of over 65% of the daily glomerular filtrate, and are among the first cells to be damaged in AKI. PTECs also play a vital role in the immune response to renal injury as the resident innate immune cells of the kidney, guiding the immune response through cytokine release in response to PRR stimulation (Carney, 2015).

In this review, we explore the role of lipotoxicity and its immunometabolic pathways in ischemic AKI. We begin by re-examining the established “direct” mechanisms of lipotoxicity within PTECs in AKI. We then explore how intracellular and extracellular lipid accumulation orchestrates the innate immune response, and how this immune response drives further lipid accumulation through PTEC metabolic reprogramming. Finally, we outline how the understanding of lipotoxicity in ischemic AKI can be used to improve clinical practice through generation of biomarkers and therapeutic targets.

### Ischemic injury causes lipid accumulation within PTECs

**FIGURE 1:**
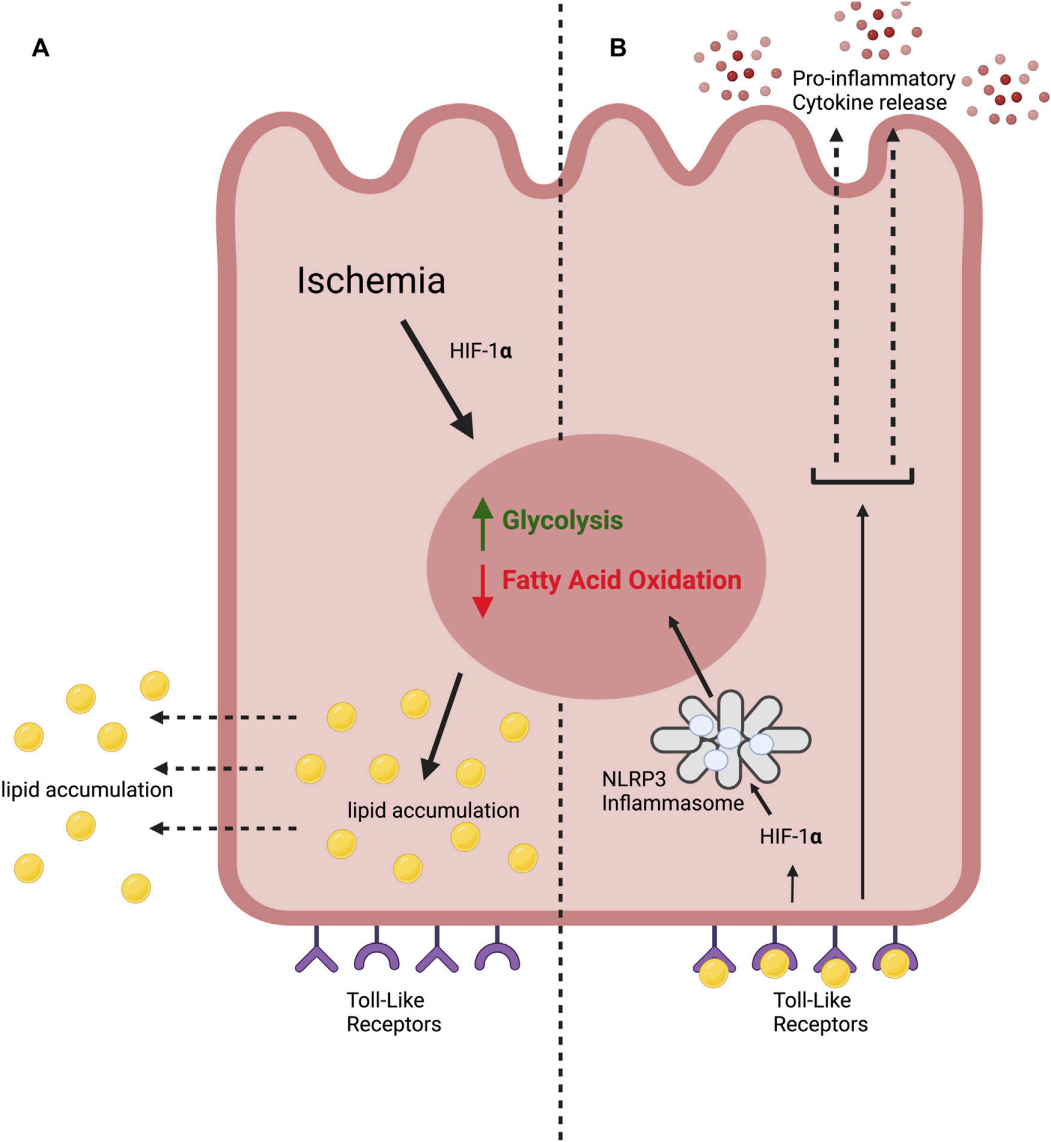
The lipotoxic effects of ischemia on the proximal tubular epithelial cell in ischemic AKI. **(A)** We hypothesise that kidney ischemia causes a “metabolic switch” in the PTEC, stimulating glycolysis and inhibiting fatty acid oxidation. This leads to lipid accumulation. **(B)** We propose that extracellular lipid accumulation stimulates Toll-like receptors (TLRs) expressed by the PTEC. This leads to the production of pro-inflammatory cytokines. TLR stimulation also leads to HIF-1α stabilisation, activating the NLRP3 inflammasome which drives glycolysis and inhibits fatty acid oxidation, compounding the metabolic switch.

AKI is associated with lipid accumulation within PTECs (Tannenbaum et al., 1983; Zager et al., 2001; Zager et al., 2005). Ischemic injury induces a metabolic switch within PTECs from fatty acid oxidation to anaerobic glycolysis with resultant accumulation of lipids (Figure 1) (Bhargava and Schnellmann, 2017). FFA’s then diffuse into the extracellular space (Scerbo et al., 2017; Jay et al., 2020).

Lipid accumulation after ischemic AKI has been demonstrated in a range of ischemia-reperfusion (IR) animal models. These experiments have followed the same format; subjecting either mice or cultured proximal tubule cells to an ischemic insult, isolating tubule cells and quantifying lipid levels using lipid dyes or enzymatic assays. However, one of the fundamental principles of lipotoxicity is that different lipids damage different cells via different mechanisms. This point was recently highlighted in the liver by Ma et al. in a mouse model of non-alcoholic fatty liver disease (Ma et al., 2016). A range of FFAs accumulated within the liver tissue, however exclusively linoleic acid was able to induce CD4 T cell death. This effect was not seen in CD8 T cells. Therefore, the attention of the field has shifted from demonstrating that there is an accumulation of FFA after ischemic injury to characterising the FFA profile.

Due to the relatively recent development of mass spectrometry techniques of sufficient resolution to identify specific fatty acids, studies presenting data on FFA profile after ischemic injury are few and far between. The studies that do exist are limited to small animal models (Table 1).

**TABLE 1:** FFA profile associated with renal ischemia reperfusion injury in small animal models.

Study	Species	Ischemic time and mechanism	Sample type	Sample time point	Controls	Measurement techniques	FFA profile	
Đurašević et al. (2019)	Rat	45 min of bilateral renal pedicle clamping	Tissue	4 h post IR	Sham operated rats	Gas chromatography mass spectrometry	Increase	Pentadecylic acid
								Palmitoleic acid
								Heptadecanoic acid
								Oleic acid
								Linoleic acid
								Docosanoic acid
								Docosahexaenoic acid
								Erucic acid
							Decrease	Myristic acid
								Palmitic acid
							No change	Stearic acid
Huang et al. (2018)	Rat	45 min of unilateral left renal artery clamping	Tissue	4 h and post IR	Contralateral kidney and kidneys from healthy control rats	Gas chromatography mass spectrometry	Increase	Palmitic acid
								Stearic acid
								Linoleic acid
								1-monopalmitin
								2-monopalmitin
Choi et al. (2019)	Rat	20 min of cardiac arrest through asphyxia, then resuscitation by cardiopulmonary bypass	Tissue	Immediately after ischemia and 30 min after reperfusion	Intact rat control	Liquid chromatography–high‐accuracy mass spectrometry	Increase	Stearic acid
								Palmitic acid
								Linoleic acid
Liu et al. (2012)	Rat	45 min of bilateral renal artery clamping	Tissue	24 h, 48 h and 96 h post-IR	Sham operated rats	Liquid chromatography–high‐accuracy mass spectrometry	Increase (24 h) (48 h)	Linoleic acid
								Arachidonic acid
								Eicosapentaenoic acid
								Stearic acid
Rao et al. (2016)	Mouse	35 min of left renal pedicle clamping	Tissue	6 and 24 h post IR	Sham operated mice	SWATH-mass spectrometry	No change (6 h)	Linoleic acid
								Arachidonic acid
Cho et al. (2017)	Mouse	45 min of left pedicle clamping	Blood	24 h post IR	Sham operated mice	Liquid chromatography–high‐accuracy mass spectrometry	Increase	Hexadecanoic acid
								Eicosenoic Acid
								Tetradecanoic acid
								Eicosatrienoic acid
								Cyclopentaneoctanoic acid
Li et al. (2022)	Mouse	22 min of left pedicle clamping	Tissue	6 h, 2 days, 7 days and 14 days post IR.	Contralateral kidney	Mass Spectrometry	Increase (6 h)	Palmitic Acid
								Oleic Acid

As shown by Table 1, whilst few studies have investigated this question, they collectively suggest that there is a unique FFA profile associated with ischemic injury, with an increase in concentration of linoleic acid, stearic acid and palmitic acid seen across multiple studies. This FFA profile persists even after cessation of ischaemic insult, with multiple studies reporting FFA elevations at both 24 and 48 h after the insult (Table 1). Further research is required to delineate the precise time course of this FFA profile, as there is a degree of disparity within the literature regarding the duration of the observed FFA signal. For example, both Huang (Huang et al., 2018) and Li (Li et al., 2022) report a reduction in FFA signal at 24 h, whereas the FFA signal in Liu’s (Liu et al., 2012) 2012 study persists for up to 48 h. We would argue that this disparity in studies can be explained by a myriad of factors. Firstly, the FFA specificity of the lipid time course, with stearic acid, demonstrated to persist for up to 48 h by Liu, not measured by either Huang or Li. In the case of FFA’s with a shorter time course, such as palmitate, Huang et al. attribute this to increased FFA catabolism, with proximal tubular cells switching to FFAs as a preferential energy source. This is reinforced by the fact that CD36 levels are upregulated post IRI (Huang et al., 2018). Whilst this reduces measurable FFAs, the lipotoxic effects of FFA are preserved as FFA are still being concentrated and uptaken by PTECs, and thus still able to act as ligands for PTEC signalling pathways. Finally, in the case of Li et al.’s 2022 study, we argue that the transient nature of the lipid accumulation reflects the fact that their IRI model is largely one of recovery, with most of their injured PT cells undergoing repair. In this model of repair, we would not anticipate observing persistent lipid accumulation; a hallmark of continued PTEC damage. Interestingly, in Li’s model of kidney injury via obstruction as opposed to ischemia, the majority of injured PTEC cells did not undergo repair but instead enter a “failed repair” state. In this model, lipids gradually accumulated over time, persisting until day 14.

Notably, this is different to the profile seen in response to ischemia in other organs, such as the liver, suggesting that this FFA profile is specific to renal ischemia (Ma et al., 2016).

Future research needs to focus on whether this FFA profile is present in humans and whether it can be used prognostically. The latter is of specific interest, as sensitive and specific biomarkers for causality and severity of AKI are still lacking^24^ .

### FFA accumulation causes direct lipotoxicity through mitochondrial dysfunction

FFA accumulation leads to dissipation of the mitochondrial membrane potential, causing mitochondrial de-energisation, dysfunction, and impaired ATP generation. FFA neutralise the mitochondrial membrane potential through two mechanisms: acting as protonophoric uncouplers and mediating mitochondrial permeability transition (MPT).

**FIGURE 2:**
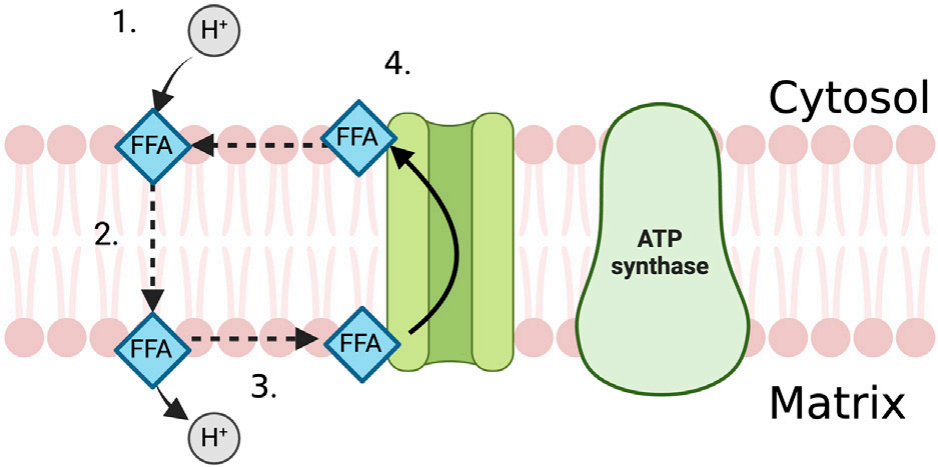
The uncoupling effect of FFA in the PTEC mitochondria. 1. FFA attracts proton on the cytosolic side of the mitochondrial membrane and becomes protonated. 2. FFA diffuses across the mitochondrial membrane in its protonated, non-polar form. 3. Proton dissociates from the FFA in the mitochondrial matrix. 4. Anion carrier transports FFA back across the mitochondrial membrane, out of the mitochondrial matrix where it can attract another proton.

In the proximal tubule, FFAs act as “uncoupling agents”, carrying protons across the inner mitochondrial membrane into the mitochondrial matrix in their protonated, non-polar form where the proton then dissociates (Feldkamp et al., 2009; Li et al., 2022). The deprotonated fatty acid ion is then shifted back across the mitochondrial membrane via anion carriers such as the adenine nucleotide translocase, the glutamate/aspartate cotransporter and the dicarboxylate and tricarboxylate carriers (Figure 2) (Weinberg et al., 2000; Feldkamp et al., 2007). This neutralises the mitochondrial membrane potential, providing protons with a route down their concentration gradient independently of ATP synthase.

In addition to providing a direct route for protons across the inner mitochondrial membrane, FFA also increase membrane permeability through MPT. MPT has recently been implicated in the pathogenesis of AKI, mediating tissue damage through mitochondria induced regulated cell death (Linkermann et al., 2012; Linkermann et al., 2013; Mulay et al., 2019). It refers to the reversible opening of the permeability transition pore (PTP), a non-specific, large conductance channel in the inner mitochondrial membrane (Bonora et al., 2022). The subsequent influx of protons, amongst other low molecular weight solutes, leads to inner membrane potential collapse and eventually the structural collapse of the organelle (Halestrap, 2009).

Whilst FFA driven PTP has not yet been demonstrated in the kidney, FFAs promote the opening of the PTP in isolated mitochondria and in liver cells (Scorrano et al., 2001; Bernardi et al., 2002; Di Paola and Lorusso, 2006). Furthermore, MPT itself is a source of fatty acids, with mitochondrial membrane swelling leading to degradation of membrane phospholipids and release of fatty acids into the cytosol (Broekemeier and Pfeiffer, 1995). This has been demonstrated in the kidney cortex, with MPT leading to the release of arachidonic and linoleic acid, both of which have been implicated by pre-clinical studies as potent uncoupling agents and inducers of MPT (Blum et al., 2011). This generates the potential for a positive feedback cycle, where FFA accumulation in ischemic AKI leads to mitochondrial membrane potential dissipation, MPT and mitochondrial damage which leads to further fatty acid accumulation.

It is important to note that the lipotoxic effects of FFA are not limited to mitochondrial dysfunction, with both lipid-mediated ferroptosis and generation of endoplasmic reticulum stress key drivers of AKI severity. Whilst important, these mechanisms are outside of the ‘immunometabolic’ scope of this review, and are comprehensively covered by Ide and Han respectively (Han and Kaufman, 2016; Ide et al., 2021).

### Mitochondrial dysfunction drives ischemic AKI

PTECs are some of the most metabolically active epithelia in the human body (Ralto and Parikh, 2016). The predominant source of ATP generation in this cell type under physiological conditions is mitochondrial oxidative phosphorylation (Ralto and Parikh, 2016). Thus, the importance of the mitochondria to the function of the proximal tubules, and therefore the kidney, cannot be overstated. It is therefore unsurprising that mitochondrial dysfunction has emerged as a ubiquitous feature of AKI, regardless of aetiology (Ralto and Parikh, 2016).

The role of mitochondrial dysfunction in AKI was first suggested in response to structural changes observed under electron microscopy in patients who had died from septic shock. The mitochondria in the proximal tubules of these patients displayed changes suggestive of organelle damage, including swelling, enlarged cristae and calcifications (Trump et al., 1975). Subsequent clinical studies taking sequential biopsies during controlled renal ischemia revealed similar lesions, further demonstrating that loss of mitochondrial ultrastructural integrity accompanies ischemic renal injury (Parekh et al., 2013). Notably, these structural changes were observed in the ischemic kidney before AKI manifested clinically, implying that mitochondrial dysfunction may contribute to the underlying pathophysiology.

These structural changes are accompanied by a myriad of functional changes. Most notably, dissipation of the mitochondrial membrane potential, increases in mitochondrial NADH, a reduction in a host of electron transport chain proteins and the reduced expression of several key mitochondrial proteins. This culminates in reduced ATP production and an energy deficit (Feldkamp et al., 2005; Funk and Schnellmann, 2012; Hall et al., 2013).

Injured mitochondria not only deprive the proximal tubule cells of ATP, but they are also a source of molecules able to induce cell death and inflammation. For example, mitochondria are responsible for around 90% of intracellular reactive oxygen species (ROS) production (Bhargava and Schnellmann, 2017). ROS are generated at low levels during normal mitochondrial metabolism and are essential in co-ordination of cellular signalling pathways. However, mitochondrial dysfunction leads to ROS overproduction, increased oxidative stress and renal injury (Havasi and Borkan, 2011; Ishimoto et al., 2018; Kaushal et al., 2019; Li et al., 2020).

Not only does mitochondrial dysfunction cause local injury, but it can cause damage to remote cell types and organs. This is mediated by the release of mitochondrial damage-associated molecular patterns (mtDAMPs). These mtDAMPS include mitochondria DNA and, when released into the circulation, activate the innate immune system (Nakahira et al., 2011). mtDAMPS generated in AKI also induce mitochondrial dysfunction in other organs, with Hepokoski et al., 2021 finding that intraperitoneal injection of renal mtDAMPs caused mitochondrial dysfunction in the lung (Hepokoski et al., 2021).

Mitochondrial dysfunction has emerged as a therapeutic target in ischemic AKI. Multiple pharmacological and cellular techniques have been demonstrated to improve mitochondrial function in preclinical models of kidney ischemic AKI (Perico et al., 2017; Szeto et al., 2017; Rousselle et al., 2020). More recently, attention has turned to mitochondrial transplantation where native, healthy mitochondria are injected into the renal arteries (Doulamis et al., 2020). Whilst promising, the translation of these studies from pre-clinical to clinical settings is significantly hampered by the lack of large animal model studies.

### FFA accumulation causes “immunometabolic” lipotoxicity through stimulation of PRRs, activating the innate immune system

**FIGURE 3:**
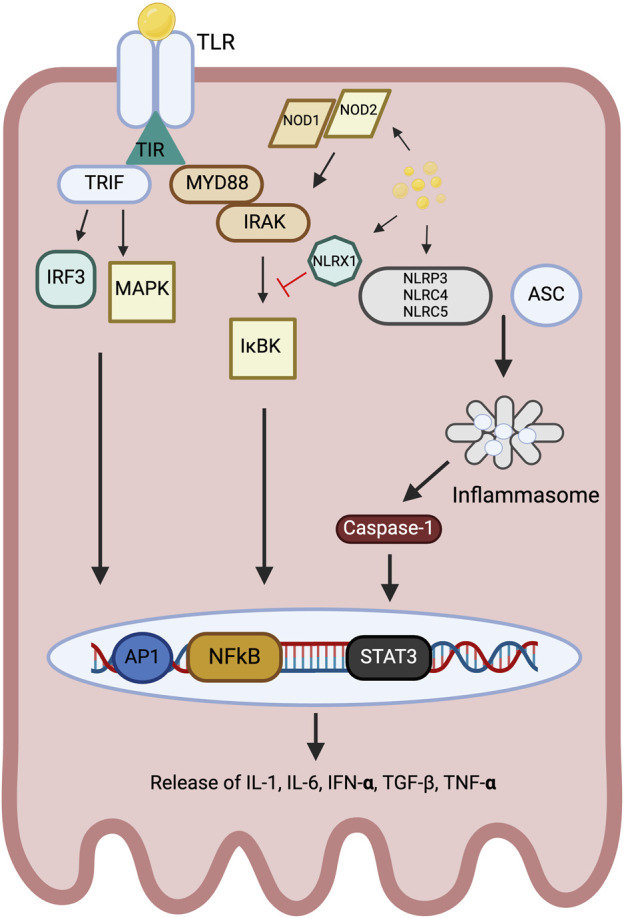
Overview of TLR and NLR signalling within the PTEC in response to intracellular and extracellular lipid accumulation.

PTECs function as the resident innate immune cells of the kidney, expressing innate immune PRRs both in the cytoplasm and on the cell membrane (Figure 3) (Deng et al., 2021). PRRs can be split into two groups, the membrane bound Toll-like receptors (TLRs) and the cytoplasmic nucleotide-binding oligomerization domain (NOD)-like receptors (NLRs). All TLRs (bar TLR3) induce the expression of inflammatory cytokines through a conserved signalling pathway, starting with the translocation of the adapter molecule myeloid differentiation factor 88 (MyD88) which ultimately leads to the early activation of nuclear factor kappa B (NFκB) (Akira and Takeda, 2004). TLR4 and TLR3 can also use an alternative signalling pathway, the MyD88-independent route, which involves the translocation of adapter molecule TRIF (TIR domain-containing adapter inducing interferon β [IFN-β]) in combination with the adapter protein TRAM (TRIF-related adapter molecule) that subsequently leads to the production of IFN-β and the expression of IFN-β-inducible genes (Figure 3) (Yamamoto et al., 2003; Rowe et al., 2006).

The intracellularly-located NLRs can be split into three subgroups, NOD1, NOD2 or NLRP based on their structure (Latz et al., 2013). NOD1 and NOD2 are sensitive to different ligands, however both act via the NF-kB and MAPK cascades, leading to the secretion of pro-inflammatory cytokines such as IL-6, IL-1β and TNF-α (Franchi et al., 2009). Activation of NLRP leads to the formation of the NLRP inflammasome in conjunction with adaptor-associated speck-like protein (ASC) and capsase-1, which in turn leads to cleavage of IL-1β and IL-18 (Figure 3) (Wen et al., 2013).

FFA activate TLR and NLR extracellularly and intracellularly, respectively. TLR signalling pathways are broadly categorised into MyD88-dependent and TRIF-dependent pathways. MyD88 forms a complex with the IRAK family, activating IkB kinase which drives NF-kB activation, whereas TRIF leads to MAP kinase activation. The NOD and NLRX1 subtypes of NLR converge on the MyD88 pathway, with NOD1 and NOD2 stimulating IRAK activation and NF-kB activation, and NLRX1 inhibiting it. NLRP3, NLRC4 and NLRC5 form an inflammasome with ASC, leading to caspase-1 activation.

These pathways lead to the activation of transcription factors including NF-kB, AP1 and STAT which drive the release of an array of pro-inflammatory cytokines.

TLRs, Toll-like receptors; NLR, Nod-like receptors; TIR, Toll/IL-1β receptor; MyD88, myeloid differentiation factor 88; IRAK, interleukin receptor-associated kinase; IκB, inhibitor of kappa B; NF-κB, nuclear factor kappa B; AP-1, activating protein-1; MAPKs, mitogen-activated protein kinase; IRF3, interferon regulatory factor 3; TRIF, TIR domain containing adaptor protein-inducing IFN-β; IL, interleukin; INF, interferon; TNF-α, tumor necrosis factor α; NLRP3, NLR family pyrin domain containing 3; NLRX1, NLR family member X1; NLRC4, NLR Family CARD Domain Containing 4; NLRC5, NLR Family CARD Domain Containing 5; ASC, apoptosis-associated speck-like protein; TGF-β, transforming growth factor-β; STAT3, Signal transducer and activator of transcription 3.

There is a growing body of evidence to suggest that PRRs are sensitive to fatty acids in a range of cell types. This interaction between the immune system and the metabolic is termed “immunometabolism”. Immunometabolism has had a significant impact on biomedicine, with aberrant cross-talk between the immunological and the metabolic implicated in disease processes across multiple organ systems (Makowski et al., 2020). We argue that immunometabolism is a fundamental and novel aspect of lipotoxicity within ischemic AKI, with TLR and NLR expression allowing PTECs to translate lipid accumulation, extracellularly and intracellularly, respectively, into an inflammatory response.

### TLRs act as cell type-specific, fatty acid-specific sensors of extracellular FFA accumulation

Multiple laboratories have independently demonstrated that FFAs exert a pro-inflammatory effect by activating TLR2, TLR3 and TLR4 (Lee et al., 2001; Senn, 2006; Nguyen et al., 2007; Schaeffler et al., 2009; Fabre et al., 2014). Since then, it has become clear that TLRs play an important role as sensors of FFAs in a range of diseases of dyslipidaemia, including atherosclerosis, diabetes and obesity (Shi et al., 2006; Rocha et al., 2016; Rogero and Calder, 2018). Whilst there is much evidence for the involvement of TLRs in FFA-sensing, there is uncertainty surrounding how FFA activate TLR signalling.

The idea that FFAs can act as direct ligands for TLRs originates from the fact that the lipid portion of lipopolysaccharide (LPS, a powerful TLR ligand) is structurally similar to saturated fatty acids (Kato et al., 1998). Docking studies investigating whether FFA can directly bind to TLRs have generated conflicting results, with Nicholas et al. using hydrophobicity protein modelling to demonstrate that palmitate could associate with the binding pocket of TLR4 in human monocyte derived dendritic cells (Nicholas et al., 2017). However, the following year, Lancaster et al. used molecular simulations to illustrate that the five palmitate molecules required to bind TLR4 would make the TLR4 active complex too unstable to function (Lancaster et al., 2018).

Whilst the evidence regarding direct binding of FFA to TLRs is still unconvincing, FFAs have been shown to induce TLR activation indirectly through two mechanisms. The first is through TLR dimerization (the first stage of TLR signalling and both pre-requisite and sufficient for activation) with lauric acid able to induce TLR4 and TLR2 dimerization in macrophages (Wong et al., 2009; Snodgrass et al., 2013). The second is through the formation of lipid rafts; plasma membrane microdomains which facilitate receptor activation through formation of receptor complexes (Wong et al., 2009; Snodgrass et al., 2013). Both of these signalling processes can occur in a ligand-independent fashion (Hwang et al., 2016). Therefore, even if FFA cannot act as a direct ligand for TLRs, their ability to modulate these indirect processes is sufficient to control TLR signalling.

The manner in which FFAs interact with these indirect processes is FFA-specific, allowing for exquisite sensitivity to the local metabolic milieu. For example, whereas lauric acid stimulates TLR2/TLR4 lipid raft formation, polyunsaturated fatty acids, in particular docosahexaenoic acid, inhibit receptor dimerization, raft formation and therefore activation (Snodgrass et al., 2013). The functional relevance of this FFA-specificity has been demonstrated in the kidney, where palmitic acid induces NF-kB-mediated inflammation and apoptosis which is inhibited by unsaturated acids oleic and eicosapentaenoic acid (Soumura et al., 2010).

### NLRs act as cell type-specific, fatty acid-specific sensors of intracellular FFA accumulation

Similarly to TLRs, there is convincing evidence to suggest that NLRs are intimately involved in the body’s inflammatory response to FFAs. Statistically significant fluctuations in the expression of NOD1, NOD2, NLRP3 and NLRX1 have been demonstrated in a range of human diseases of dyslipidaemia, including, obesity, diabetes, non-alcoholic fatty liver disease, metabolic syndrome and atherosclerosis (Schertzer et al., 2011; Vandanmagsar et al., 2011; Cuda et al., 2012; Freigang et al., 2013; Wang et al., 2013; Zhou et al., 2015; Kors et al., 2018; Cavallari et al., 2020). These diseases are characterised by deviations in a range of metabolites (including glucose and amino acids), therefore do not provide the required resolution to draw conclusions on FFA-sensing. However, the observed clinical associations have acted as a catalyst for research exploring the role of NLRs as sensors of FFA. These studies have demonstrated that FFAs can activate NLRs both directly and indirectly. For example, the C-terminus of NLRX1 can directly bind a range of unsaturated fatty acids (Lu et al., 2015), whereas FFA interact with NLRP3 indirectly by activating the inflammasome via the AMPK pathway (Karasawa et al., 2018).

Saturated and unsaturated fats play opposing, specific, roles in the regulation of NLR. Whilst saturated fats activate the NLRP3 inflammasome in macrophages and adipocytes, monounsaturated fats either counteract this activation or even downregulate NLPR3 (Wen et al., 2011; L’homme et al., 2013; Finucane et al., 2015). This can also be observed in NOD1 and NOD2. Saturated fatty acids stimulate receptor activation and pro-inflammatory cytokine release in intestinal epithelial cells and adipocytes, whereas unsaturated fatty acids impair NOD1 and NOD2 receptor activation (Zhao et al., 2007; Zhou et al., 2013).

The resolution of this FFA-specific NLR signalling extends beyond saturated versus unsaturated, and is cell type-specific. This is best seen in the interaction of the NLRP3 inflammasome with the short chain fatty acids acetate, propionate and butyrate. Acetate decreased NLRP3 inflammasome in lung tissue, however stimulates NLRP3 activation in intestinal epithelial cells (Macia et al., 2015; Zhang et al., 2021). Propionate inhibits NLRP3 activation in intestinal epithelial cells, however stimulates NLPR3 activation in intestinal macrophages (Tsugawa et al., 2020; Yang et al., 2020). Butyrate also stimulates NLPR3 activation in intestinal macrophages, however inhibits NLPR3 inflammasome formation in corneal epithelial cells (Bian et al., 2017; Tsugawa et al., 2020).

### PRR activation drives ischemic AKI through generation of a pro-inflammatory, pro-apoptotic response

PRRs are constitutively expressed in PTECs and have been demonstrated to play a role in ischemia reperfusion injury (IRI) animal models of ischemic AKI (Leemans et al., 2005; Pulskens et al., 2008; Paulus et al., 2014; Perico et al., 2017). TLR2, TLR3 and TLR4 mRNA are upregulated upon IRI in mice (Wolfs et al., 2002; Paulus et al., 2014), and TLR2 and TLR4 over-expression in PTECs exposed to IRI injury leads to an amplified inflammatory response and impaired renal function (Wu et al., 2007; Leemans et al., 2014). In contrast, TLR2, TLR4 and TLR3 knockout (KO) mice are protected against IRI compared to WT controls (Leemans et al., 2005; Pulskens et al., 2008; Paulus et al., 2014). Shigeoka et al. demonstrated that NOD2 KO and NOD1/NOD2 double KO mice were protected from renal tubular apoptosis and inflammation compared to wild type in IRI (Shigeoka et al., 2010). Similar protection against renal damage in response to ischemia has been seen in NLRP3 KO mice (Iyer et al., 2009; Kim et al., 2013), NLCR5 KO mice and NLCR4 suppressed mice (Guo et al., 2018Guo et al., 2018).

These receptors play specific roles within the pathophysiology of ischemic AKI. For example, TLR2 and TLR4 are activated much later than TLR3, which is activated within minutes of ischemic insult. This was demonstrated by Paulus et al., in 2014, who hypothesised that TLR3 acts as a ‘trigger mechanism’ involved in cross talk with the other TLRs (Paulus et al., 2014). Furthermore, TLR2 and TLR3 drive the apoptotic processes seen in ischemic AKI, whereas TLR4 plays a more pro-inflammatory role through stimulating chemokine release and granulocyte migration (Leemans et al., 2005; Pulskens et al., 2008; Paulus et al., 2014).

NLRs also play specialised roles within ischemic AKI pathophysiology. NOD1 and NOD2 appear to mediate their effect through stimulation of apoptosis (Shigeoka et al., 2010), whereas NLPR3 and NLCR5 play a more directly pro-inflammatory role through release of cytokines, and immune cell infiltration and activation (Kim et al., 2013; Li Q. et al., 2018). NLRX1 plays a protective role in ischemic AKI, with NLRX1 KO leading to more severe mitochondrial damage, oxidative stress and renal epithelial cell apoptosis in an IRI mouse model (Stokman et al., 2017). This protection occurs through a reduction of mitochondrial oxidative stress at the level of the mitochondrial permeability transition pore, with treatment of NLRX1 knock out mice with cyclosporine A (an inhibitor of MPTP opening) reducing apoptosis compared to normoxic controls. This provides a point of convergence between the direct mechanisms of lipotoxicity described earlier in this review and immunometabolic lipotoxicity, with fatty acids able to modulate the MPTP either directly or through PRR activation. For example, docosahexaenoic acid, a ligand of NLRX1, reduces PTEC MPT and apoptosis, whereas palmitic acid—which is not a ligand of NLRX1—directly induces cell MPT and apoptosis (Scorrano et al., 2001; Jiang et al., 2022).

### PRRs translate lipid accumulation into a pro-inflammatory, pro-apoptotic immune response within the ischemic kidney

Whilst both TLRs and NLRs are associated with ischemic AKI pathophysiology, this has not yet been explored as a response to lipid accumulation. The evidence that does exist is sparse and focuses on kidney dysfunction as a complication of systemic diseases of dyslipidaemia, such as obesity and diabetes, as opposed to primary kidney disease.

In the case of diabetes, these findings represent correlation at best. Inhibition of TLR4 or NOD2 has been demonstrated to reduce renal injury and inflammation in Streptozotocin mouse models of diabetic nephropathy (Du et al., 2013; Lin et al., 2013). Plasma FFA are raised in these mouse models, therefore this anti-inflammatory effect could represent a reduction in FFA-mediated TLR4 or NOD2 activation. However, it is difficult to draw any conclusions due to the range of metabolites besides FFA which could stimulate NLR and TLR and therefore act as confounders. For example, hyperglycaemia is a known activator of NOD2 and TLR4 (Lim and Tesch, 2012; Wang et al., 2015).

Obesity induced nephropathy provides a more robust model of PRRs as sensors of lipid accumulation in kidney injury. For example, Li et al., in 2018 used a high fat diet mouse model of obesity-induced nephropathy to demonstrate palmitate-mediated renal tubular inflammation and apoptosis via the NLPR3/Caspase-1 axis (Li L-C. et al., 2018). This occurred in a CD36-dependent fashion; a scavenger receptor known to mediate intracellular uptake of FFA in acute kidney injury. Kors et al., 2018 used a similar high fat diet mouse model, finding that NLRX1 KO mice exhibited less renal lipid accumulation compared to wild type, and were subsequently protected against western diet-induced nephropathy (Kors et al., 2018).

TLR antagonism using GIT27 protects mice against high fat diet-induced kidney disease through suppression of pro-inflammatory chemokine release and immune cell migration (Min et al., 2014). The authors suggested that this was likely through inhibition of FFA-stimulated TLR4 signalling, as podocyte culture in palmitate solution stimulated NFκB and IκBβ production, both of which were inhibited significantly by GIT27.

Regarding ischemic AKI, only one study has investigated the role of lipid activated PRRs in renal injury. Jeon et al. explored the role of a high fat diet in an IRI mouse model, reporting that mice receiving a high fat diet were more susceptible to ischemic injury (Jeon et al., 2021). This was attributed to increased intrarenal CD8 T cells and plasma cell numbers, secondary to increased expression of TLR2 and TLR4. This provides a mechanism wherein immunometabolic pathways drive lipotoxicity in ischemic AKI, as fatty acid accumulation is sensed by TLRs and translated into a nephrotoxic immune response.

Whilst this is promising, it is important to acknowledge that these studies all focus on exogenous FFA provided by a high fat diet. These high fat diet models are likely to generate a higher concentration of extracellular lipids than those seen in ischemic kidney injury, and there is currently no direct evidence to suggest that free intracellular or extracellular FFAs reach concentrations sufficient to compete with other ligands for PRRs in the context of ischaemic kidney injury. Therefore, future research needs to investigate whether the endogenous FFA produced via the mechanisms outlined previously in this review are capable of generating a similar immunometabolic, lipotoxic response.

### PRRs drive further lipid accumulation, generating an ischemia-independent positive feedback cycle of lipotoxicity

Thus far in this review, when describing immunometabolic lipotoxicity we have referred to the ability of PPRs to sense the metabolic microenvironment and translate this into an inflammatory response. However, PRRs also modulate immune cell metabolism in response to the metabolic microenvironment.

Physiologically, this aspect of immunometabolism likely acts as a negative feedback mechanism to correct external metabolic aberrations or to generate pro-inflammatory immune cell phenotypes able to respond to the cause of DAMP production. In the ischemic kidney, however, PRR activation perpetuates the ‘metabolic switch’ from fatty acid oxidation to glycolysis (Figure 1) stimulated by the original ischemic injury, leading to further lipid accumulation.

**FIGURE 4:**
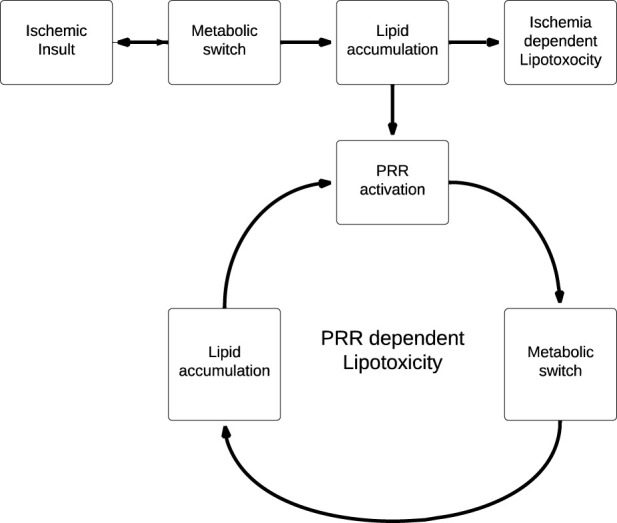
Linear (ischemia dependent) vs. cyclical (PRR dependent) models of lipotoxicity. PRR dependent lipotoxicity generates a positive feedback cycle, where lipid accumulation is no longer coupled to the original ischemic insult.

This has significant implications for the role of lipotoxicity in ischemic AKI, as PRR activation by lipid accumulation induced by the initial ischemic insult leads to further lipid accumulation which, in turn, leads to further PRR stimulation (Figure 4). We propose that this generates a positive feedback cycle able to perpetuate the effects of lipotoxicity beyond the original ischemic insult, as lipid accumulation is now PRR-dependent and ischemia-independent. Of course, it is important to note that this model is speculative, and limited by the fact that there is as of yet no clear mechanism to halt the positive feedback cycle.

### PRR activation drives lipid accumulation through stimulating a metabolic switch in PTECs

Currently there is no direct data on the role of PRRs in metabolic switching in the ischemic kidney. Indeed, evidence for a role of PRRs in modulation of metabolic pathways in any form of kidney pathology is limited. Wu used the NLRP3-specific inhibitor MCC950 and NLRP3 KO mice to demonstrate that NLRP3 stimulates fatty acid synthesis in diabetic nephropathy through increased expression of the fatty acid synthesis genes SREBP1 and SREBP2 (Wu et al., 2021). Using the same techniques, Rampanelli demonstrated that NLPR3 inhibited fatty acid oxidation in obesity-induced nephropathy through disruption of the SIRT1/LKB1/AMPK axis (Rampanelli et al., 2017).

Evidence for the role of TLRs in metabolic switching in the kidney comes from Kenneth et al., who found that treatment of mice with the TLR4 ligand LPS led to a 40% reduction in kidney fatty acid oxidation through PPARα suppression, as well as other genes involved in fatty acid oxidation (Feingold et al., 2008). Furthermore, TLR4 has been demonstrated to upregulate the glycolytic enzyme hexokinase in the kidney (Robey et al., 2002; Smith et al., 2014).

Beyond the kidney, there is a wealth of evidence in a range of cell types to support the role of PRRs as controllers of a cell’s metabolic phenotype (Krawczyk et al., 2010; Kelly and O'Neill, 2015; Singh et al., 2015; Singh et al., 2019; Guo et al., 2021). This is especially well characterised in immune cells, where metabolic phenotype heavily influences cell activation and polarisation (Chou et al., 2022). For example, in myeloid cells, it is well documented that TLR2 and TLR4 activation stimulate glycolysis and inhibit fatty acid oxidation, whereas NLRX1 promotes glycolysis in CD4^+^ T cells (Krawczyk et al., 2010; Everts et al., 2014; Kelly and O'Neill, 2015; Guo et al., 2021).

Hepatocytes provide another example of the power of PRRs as metabolic controllers. This has been extensively investigated in the context of non-alcoholic steatohepatitis (NASH) and non-alcoholic fatty liver disease (NAFLD), where the switch to a metabolic phenotype favouring glycolysis and fatty acid synthesis leads to the characteristic lipid deposition (Lu et al., 2021).

In NASH/NAFLD, PRRs have two effector mechanisms to facilitate hepatocyte metabolic switch. The first is through cytokine release, with TLRs and NLRs expressed on the surface of hepatocytes and Kupffer cells stimulating the release of IL-1β and TNF-α (Negrin et al., 2014; Khanmohammadi and Kuchay, 2022), which leads to the increased expression of genes involved in fatty acid synthesis such as SREBP1 (Todoric et al., 2020). The second is through direct action on the hepatocyte, with the TLR4/TRIF signalling pathway and the NLRX1/AMPK axis recently demonstrated to modulate DGAT2 and AMPK activity, respectively; two enzymes fundamental in fatty acid metabolism (Yang et al., 2017; Kors et al., 2018).

Whilst future research needs to focus on establishing whether similar mechanisms are at play in the ischemic kidney, parallels already exist within the literature. For example, both IL-1β and TNF-α are released in high concentrations in IRI, and IL-1β is known to promote renal steatosis in models of diabetic nephropathy through upregulation of SREBP1 in an NLRP3-dependent fashion (Wang C. et al., 2012; Wu et al., 2021).

### PRR-mediated metabolic switch in the ischemic kidney is driven by a TLR4/HIF/NLRP3 axis

The link between PRR activation and the metabolic switch in the ischemic kidney could be the ubiquitous transcription factor HIF1-α. The potential for HIF-1α to drive renal lipid accumulation through metabolic reprogramming of PTECs can be modelled using clear cell renal carcinoma. Sporadic clear cell carcinoma is a PTEC cancer characterised by a loss of function mutation in the von Hippel-Lindau (VHL) protein, which is essential for HIF-1α degradation (Cowey and Rathmell, 2009). This causes upregulated HIF-1 transcriptional activity and HIF-1-dependent metabolic reprogramming, stimulating a shift towards glycolysis and a shift away from fatty acid catabolism through transcriptional regulation of enzymes, such as CPT1A and hexokinase, transporters including GLUT1, and binding proteins such as FABP3 (Chan et al., 2011; Bensaad et al., 2014; Du et al., 2017; Yang et al., 2017; Zhang Y. et al., 2021). This HIF-dependent metabolic switch then drives lipid accumulation, a hallmark of renal clear cell carcinoma (van der Mijn et al., 2019).

We propose that HIF-1α plays a similar role in the metabolic rewiring seen in the ischemic kidney. However, instead of the VHL loss of function mutation, HIF-1 α accumulation is driven by TLR4 signalling as part of a TLR4/HIF-1α/NLRP3 axis. TLR4 activation induces HIF-1 accumulation, which acts through NLRP3 to drive lipid accumulation through stimulation of glycolysis and fatty acid synthesis with inhibition of oxidative phosphorylation and fatty acid oxidation.

HIF-1 α accumulation in response to TLR4 activation by LPS has been demonstrated in a range of cell types (Ramanathan et al., 2009; Zhou et al., 2014; Yang et al., 2018), both directly through activation of the HIF-1α promotor region, and indirectly by downregulation of HIF degradation by prolyl hydroxylases or activation of lipid rafts (Frede et al., 2006; Nicholas and Sumbayev, 2010; Yang et al., 2018). As with TLR4-mediated HIF-1 accumulation, there is convincing evidence of HIF-1-mediated activation of NLRP3 outside of the kidney. For example, Hernandez et al. demonstrated that elevated HIF-1α expression promotes NLRP3/inflammasome/caspase-1 activation in fat-laden rat hepatocytes (Hernández et al., 2020), and Ouyang found that LPS-primed HIF-1α KO murine macrophages had significantly less NLPR3 inflammasome activation than wild type (Ouyang et al., 2013). This effect was also seen when macrophages were treated with the HIF-1α inhibitor CAY10585.

The role of a TLR4/HIF1/NLRP3 axis in the ischemic kidney has not been well characterised. Sun et al. demonstrated that HIF-1α stimulates NLPR3 activation in a rat IRI model (Sun et al., 2020), however did not measure TLR4 expression or use LPS as a ligand and, therefore, there is no direct evidence to suggest that this is TLR-mediated. Zhang, in a porcine IRI model, found that both TLR4 and HIF-1 were overexpressed in response to hypoxia, however identifying any interactions between the two molecules was reported as beyond the scope of the study (Zhang et al., 2016). Future research needs to focus on identifying this pathway in the ischemic kidney. Beyond this, it is important to note that all evidence thus far has focused on the pro-inflammatory effects of NLRP3 activation, whereas future research must also focus on identifying the metabolic effects of TLR4/HIF-1/NLRP3 activation. Given the ubiquity of this pathway, this line of research could have significant implications for the pathophysiology of lipotoxicity across multiple organ systems.

### Future perspectives: lipid biomarkers in AKI

The central role of lipotoxicity in ischemic AKI pathophysiology has important implications for clinical practice. The first of which is using lipids as sensitive biomarkers for early detection and prognostication of AKI, and the second is using lipid modulating drugs to improve outcomes.

One of the major limitations of AKI research has been the inability to identify a ‘kidney troponin’; that is, a sensitive and specific biomarker to allow early detection and prognostication of kidney injury. Traditionally, serum creatinine and urine output have been used to define and prognosticate AKI. However, these markers are heavily flawed, limited by low sensitivity, low specificity and delayed changes, with creatinine taking 24–36 h to rise following kidney injury (Zou et al., 2022). Whilst several new biomarkers have been identified, none have reached sufficient specificity or sensitivity.

This has had significant implications in clinical practice, where the early detection of AKI is essential for timely and effective initiation of preventative management, and in research, where the tardiness of experimental interventions is often blamed for the lack of novel therapeutics (Alge and Arthur, 2015). The surge in proteomics research in the early 2000s fostered the identification of a new wave of protein biomarkers in plasma, urine and tissues which preceded changes in classical renal biomarkers (Devarajan, 2007; Portilla et al., 2007). We argue that the ongoing surge in lipidomics and metabonomics could yield similar results, as ischemic AKI is associated with a rapid and specific rise in lipid (and lipid metabolite) concentration, with the magnitude and composition of this lipid accumulation correlated with AKI severity.

It is not known whether a similar lipid profile exists in humans, however research into fluctuations of plasma, urine and tissue lipids in response to AKI could generate a host of novel biomarkers. The potential utility of lipid-based biomarkers in humans was demonstrated by Jonson et al., 2011, who found that urinary cholesterol levels in critically ill patients with AKI were doubled compared to critically ill patients without AKI (Johnson et al., 2011). Interestingly, urine pellet cholesterol values were normal in patients with CKD, suggesting a level of specificity that many of the novel proteomic biomarkers, such as NGAL, KIM-1 and MCP-1, do not possess.

### Lipotoxicity: therapeutic targets

As the appreciation of the importance of lipotoxicity in disease pathophysiology has grown, so has interest in targeting it therapeutically. Lipid lowering pharmacological interventions are currently being trialled in a range of organ systems and disease processes, from atherosclerosis to fatty liver disease (Gupta et al., 2019; Neuschwander-Tetri, 2020).

Investigations into the efficacy of lipid lowering medication in acute kidney injury is still in its infancy. These preclinical studies have focused on two main lipid lowering drugs: PPAR-α agonists and SGLT2 inhibitors.

Since Li demonstrated that PTEC PPAR-α overexpression in mice conferred protection against IRI by maintaining fatty acid oxidation, PPAR-α agonists have been proposed for therapeutic use (Li et al., 2009). PPAR-α agonists clofibrate, fenofibrate and WY1463 all reduced renal dysfunction in rat models of IRI, an effect lost in PPAR-α KO (Portilla et al., 2000; Sivarajah et al., 2002). These medications enhance lipolysis by overexpressing CPT-1, acyl-coA oxidase, and medium-chain acyl-CoA dehydrogenase (Tanaka et al., 2011).

SGLT2 inhibitors represent a newer wave of anti-lipotoxic medications, with recent studies finding that SGLT2 inhibitors decrease hepatic fat in patients with type 2 diabetes (Scheen, 2019; Sabine et al., 2020). Whether SGLT2 inhibitors have a similar effect in the kidneys has yet to be explored in humans, however SGLT2 inhibition has been shown to reduce lipid accumulation and improve IRI outcomes in several mouse models (Xiaoxin et al., 2017; Kohshiro et al., 2019; Ala et al., 2022).

Whilst these results are promising, further large scale, prospective, randomised trials are required to evaluate the effects of lipid lowering medications in patients with AKI.

## Conclusion

In conclusion, lipid accumulation and toxicity are cardinal features of ischemic AKI. This lipotoxicity acts via two mechanisms, directly through mitochondrial dysfunction and subsequent tubule cell apoptosis, and indirectly through innate immune system-mediated inflammation. The immunometabolic pathways driving the latter represent a new paradigm through which the effects of lipotoxicity can be understood, with FFAs able to not only generate an inflammatory response through activation of PRRs, but also contribute to lipid accumulation through metabolic reprogramming of tubular cells. Future research must focus on characterising these immunometabolic pathways, to better our understanding of AKI and ultimately improve its management.
