# Supplementary material for: Lipotoxicity and immunometabolism in ischemic acute kidney injury: current perspectives and future directions
Source: Front Pharmacol. 2024 Feb 23;15:1355674. doi: 10.3389/fphar.2024.1355674 (PMC10924325; doi:10.3389/fphar.2024.1355674)
Supplement: Supplementary file 1 [file Table2.docx]

| **Receptor Class** | **Receptor Name** | **Function in Ischemic AKI** | **FFA Ligand** | **Target** | **Action** |
| --- | --- | --- | --- | --- | --- |
| TLR | TLR2 | Pro-inflammatory via neutrophil infiltration, chemokine release (KC, MIP-2, MCP-1) and cytokine release (IL-6 and IL-1β)  Pro-apoptotic  Pro-necrotic  (Leemans 2005^65^) | Palmitic Acid | 293T embryonic kidney cells (Human) | Activation of MyD88/NFκB signalling pathways (Lee 2004^66^) |
|  |  |  |  | Skeletal muscle myotubes (mouse) | Increased insulin resistance  (Senn 2006^67^) |
|  |  |  |  | THP-1 monocytes (Human) | Increased IL-1β secretion  (Snodgrass 2013^68^) |
|  |  |  |  | Primary heart endothelial cells (Mouse) | Increased E-selectin, TNFα, IL-6 and IL-1β expression (Jang 2013^69^) |
|  |  |  |  | BV2 Microglia (Mouse) | Reduced Microglia TNFα production and increased NO production, no effect on IL-6 production  (Howe 2022^70^) |
|  |  |  | Lauric Acid | RAW264.7 Macrophages (Mouse) | COX-2 expression with induction of IL-1α and iNOS (Lee 2004^66^) |
|  |  |  | Docosahexaenoic acid (U) | RAW264.7 Macrophages (Mouse) | Inhibition of COX-2 expression  (Lee 2003^71^) |
|  |  |  |  | Dendritic cells (Mouse) | Reduction in IL-12 and IL-23 production (Kong 2010^72^) |
|  |  |  | Oleic Acid | BV2 Microglia (Mouse) | Reduced Microglia IL-6 production, increased iNOS expression with no effect on TNF-α (Howe 2022^70^) |
|  | TLR3 | Pro-inflammatory via neutrophil infiltration  Pro-apoptotic  Pro-necrotic  Initiation of TLR2/TLR4 response  (Paulus 2014^73^) | Palmitic Acid | Skeletal muscle myotubes (Mouse and Human) | Increased insulin resistance  (Fabre 2014^74^) |
|  |  |  | Docosahexaenoic acid (U) | Dendritic cells (Mouse) | Reduction in IL-12 and IL-23 production (Kong 2010^72^) |
|  | TLR4 | Pro-inflammatory via neutrophil infiltration and chemokine release (KC)  Pro-necrotic  (Pulskens 2008 ^75^) | Palmitic Acid | RAW264.7 Macrophages (Mouse) | COX-2 expression with induction of IL-1α and iNOS (Lee 2001 ^76^) |
|  |  |  |  | Adipocytes (Mouse) | Increased Adipokine secretion  (Schaeffler 2009 ^77^) |
|  |  |  |  | Skeletal muscle myotubes (Mouse and Human) | Increased insulin resistance  (Fabre 2014 ^74^) |
|  |  |  |  | Aortic vascular smooth muscle cells (Human) | Increased IL-8 expression  (Quan 2014^78^) |
|  |  |  |  | Astrocytes (Rats) | Increased TNF-α and IL-6 expression (Gupta 2012^79^) |
|  |  |  |  | BV2 Microglia (Mouse) | Il-6, TNF-α and IL-1β expression (Wang 2012 ^80^) |
|  |  |  |  | MIN6 Beta-islet cells (Mouse) | MCP-1 and KC expression  (Eguchi 2012^81^) |
|  |  |  | Lauric Acid | RAW264.7 Macrophages (Mouse) | Stimulation of COX-2 expression with induction of IL-1α and iNOS (Lee 2001 ^76^) |
|  |  |  | Stearic Acid | Adipocytes (Mouse) | Increased Adipokine secretion (resistin and MCP-1)  (Schaeffler 2009^77^) |
|  |  |  |  | BV2 Microglia (mice) | Il-6, TNF-α and IL-1β expression (Wang 2012 ^80^) |
|  |  |  | Palmitoleic Acid | Adipocytes (Mouse) | Increased Adipokine secretion (resistin)  (Schaeffler 2009 ^77^) |
|  |  |  | Docosahexaenoic acid (U) | RAW264.7 Macrophages (Mouse) | Inhibition of COX-2 expression  (Lee 2003^71^) |
|  |  |  |  | Intestinal microvascular endothelial cells (Human) | Inhibition of COX-2 expression, reduction in production of IL-6, IL-8 and PGE2 (Ibrahim 2011 ^82^) |
|  |  |  |  | Dendritic cells (Mouse) | Reduction in IL-12 and IL-23 production (Kong 2010 ^72^) |
|  |  |  |  | Breast cancer tissue (Human) | Induction of cell apoptosis  (Geng 2018 ^83^) |
|  |  |  |  | Skeletal muscle C2C12 myotubes | Attenuation of palmitate induced insulin resistance (Yu 2015 ^84^) |
|  |  |  | Eicosapentaenoic acid (U) | RAW264.7 Macrophages (Mouse) | Inhibition of COX-2 and PGE2 expression, reduction in production of IL-2, IL-1 and TNF-α (Lee 2003 ^71^) |
| NLR | NOD1 | Pro-Apoptopic  (Shigeoka 2010) | Lauric Acid | Colonic epithelial HCT116 cells (Human) | Increased IL-8 production  (Zhao 2007^85^) |
|  |  |  | Docosahexaenoic acid (U) | Colonic epithelial HCT116 cells (Human) | Inhibited IL-8 production  (Zhao 2007 ^85^) |
|  |  |  | Eicosapentaenoic acid (U) | Colonic epithelial HCT116 cells (Human) | Inhibited IL-8 production  (Zhao 2007 ^85^) |
|  | NOD2 | Pro-Apoptopic  (Shigeoka 2010^86^) | Lauric Acid | Colonic epithelial HCT116 cells (Human) | Increased IL-8 production  (Zhao 2007 ^85^) |
|  |  |  | Docosahexaenoic acid (U) | Colonic epithelial HCT116 cells (Human) | Inhibited IL-8 production  (Zhao 2007 ^85^) |
|  |  |  | Eicosapentaenoic acid (U) | Colonic epithelial HCT116 cells (Human) | Inhibited IL-8 production  (Zhao 2007 ^85^) |
|  | NLRP3 | Pro-Inflammatory via neutrophil infiltration, cytokine release (IL-6 and IL-1) and chemokine release (CXCL1)  Pro-Apoptopic  (Kim 2013^87^,  Iyer 2009 ^88^) | Palmitic acid | Bone marrow derived macrophages (Mouse) | Increased IL-1β and Caspase-1 production  (Wen 2011^89^) |
|  |  |  |  | Sw.71 Placental Cells (Human) | Increased ROS production and IL-1β, IL-6 and IL-8 secretion.  (Shirasuna 2016^90^) |
|  |  |  |  | Primary Hepatocytes (Mouse) | Increased IL-1β and Caspase-1 production  (Csak 2011^91^, Sui 2016^92^) |
|  |  |  |  | Primary Macrophages (Mouse) | Increased IL-1β and Caspase-1 production (Karasawa 2018^93^) |
|  |  |  |  | HepG2 hepatoma cells (Human) | Induction of IL-1β-mediated pyroptosis (Zeng 2020 ^94^) |
|  |  |  | Stearic Acid | Primary Macrophages (Mouse) | Increased IL-1β and Caspase-1 production (Karasawa 2018^93^) |
|  |  |  | Oleic Acid  (U) | Primary Macrophages (Mouse) | Reduced IL-1β and Caspase-1 production (Karasawa 2018^93^) |
|  |  |  |  | HepG2 hepatoma cells (Human) | Inhibition of palmitate induced of IL-1β-mediated pyroptosis  (Zeng 2020 ^94^) |
|  |  |  | Docosahexaenoic acid (U) | Primary Hepatocytes (Mouse) | Inhibited IL-1β and Caspase-1 production  (Csak 2011^91^, Sui 2016^92^) |
|  |  |  |  | THP-1 monocytes (Human) | Inhibition of IL-1β and Caspase-1 production (Yan 2013, Williams-Bey 2014) |
|  |  |  | Eicosapentaenoic acid (U) | THP-1 monocytes (Human) | Inhibition of IL-1β and Caspase-1 production  (Yan 2013) |
|  | NLRX1 | Anti-apoptotic via reduction of mitochondrial oxidative stress  Anti-inflammatory via reduced secretion of chemokines (CXCL1 and CCL2)  (Stokman 2017^95^) | Punicic acid (U) | Colonic epithelium (Mouse) | Reduced epithelial erosion, leukocytic infiltration and mucosal thickening (Lu 2015 ^96^) |
|  |  |  |  | Bone marrow derived macrophages (Mouse) | Anti-inflammatory effects through suppression of NF-kB activity  (Lu 2015 ^96^) |
|  |  |  | Docosahexaenoic acid (U) | Bone marrow derived macrophages (Mouse) | Anti-inflammatory effects through suppression of NF-kB activity  (Lu 2015 ^96^) |
|  | NLRC4 | Pro-Inflammatory via cytokine release (TNF-α, IL-18, CXCL1 and CXCL2) and macrophage infiltration.  Pro-apoptopic  (Guo 2017^97^) | Palmitic Acid | Primary Astrocytes (Rat) | Increased IL-1β and Caspase-1 production  (Liu 2014^98^) |
|  |  |  |  | HepG2 hepatoma cells (Human) | Increased IL-1β, IL-18, TNF-α, and MCP-1 secretion.  (Luo 2012^99^) |
|  |  |  | Docosahexaenoic acid (U) | THP-1 monocytes (Human) | Inhibition of IL-1β and Caspase-1 production (Williams-Bey 2014^100^) |
|  |  |  |  | HepG2 hepatoma cells (Human) | Inhibition of palmitate-mediated IL-1β, IL-18, TNF-α, and MCP-1 secretion.  (Luo 2012^99^) |

**Table 2. Pattern recognition receptors of the innate immune system and their fatty acid ligands.**
